# Sequence and phylogenetic analysis of novel porcine parvovirus 7 isolates from pigs in Guangxi, China

**DOI:** 10.1371/journal.pone.0219560

**Published:** 2019-07-10

**Authors:** Wei Wang, Liang Cao, Wenchao Sun, Jialiang Xin, Min Zheng, Mingyao Tian, Huijun Lu, Ningyi Jin

**Affiliations:** 1 College of Animal Science and Technology, Guangxi University, Nanning, People’s Republic of China; 2 Institute of Military Veterinary, Key Laboratory of Jilin Province for Zoonosis Prevention and Control, Academy of Military Sciences, Changchun, People’s Republic of China; 3 College of Animal Science and Technology, Jilin Agricultural University, Changchun, People’s Republic of China; 4 Institute of Virology, Wenzhou University, Wenzhou, People’s Republic of China; 5 Guangxi Center for Animal Disease Control and Prevention, Nanning, People’s Republic of China; University of Kansas Medical Center, UNITED STATES

## Abstract

Parvoviruses are a diverse group of viruses that infect a wide range of animals and humans. In recent years, advances in molecular techniques have resulted in the identification of several novel parvoviruses in swine. In this study, porcine parvovirus 7 (PPV7) isolates from clinical samples collected in Guangxi, China, were examined to understand their molecular epidemiology and co-infection with porcine circovirus type 2 (PCV2). In this study, among the 385 pig serum samples, 105 were positive for PPV7, representing a 27.3% positive detection rate. The co-infection rate of PPV7 and PCV2 was 17.4% (67/385). Compared with the reference strains, we noted 93.9%-97.9% similarity in the *NS1* gene and 87.4%-95.0% similarity in the *cap* gene. Interestingly, compared with the reference strains, sixteen of the PPV7 strains in this study contained an additional 3 to 15 nucleotides in the middle of the *cap* gene. Therefore, the Cap protein of fourteen strains encoded 474 amino acids, and the Cap protein of the other two strains encoded 470 amino acids. However, the Cap protein of the reference strain PPV7 isolate 42 encodes 469 amino acids. This is the first report of sequence variation within the *cap* gene, confirming an increase in the number of amino acids in the Cap protein of PPV7. Our findings provide new insight into the prevalence of PPV7 in swine in Guangxi, China, as well as sequence data and phylogenetic analysis of these novel PPV7 isolates.

## Introduction

The family *Parvoviridae* is classified into two subfamilies, *Parvovirinae* and *Densovirinae*, whose hosts are vertebrates and arthropods, respectively [1,2]. Most members of the subfamily *Parvovirinae* cause only mild clinical symptoms, but a small number are causative agents of important diseases, for example, goose parvovirus (geese: Gosling Plague), porcine parvovirus 1 (pigs: mainly reproductive disorders) and parvovirus B19 (humans: infectious erythema) [2,3]. Parvoviruses are small, single-stranded linear, non-enveloped DNA viruses with a genome of approximately 4–6 kb [2]. The genome contains two major open reading frames (ORFs) [4]. ORF1 encodes non-structural proteins (NS) involved in viral replication, while ORF2 encodes structural (Cap) proteins [5]. An additional ORF, ORF3, encodes nuclear phosphoproteins (NP) and is located in the middle of ORF1 and ORF2. It is characteristic of members of the *Bocaparvovirus* genus [6,7].

To date, six porcine parvovirus (PPV) genotypes (PPV1-6) have been discovered [8,9]. According to the amino acid similarity in the NS1 protein, these viruses are taxonomically divided into three genera [2]: *Protoparvovirus* (PPV1), *Tetraparvovirus* (PPV2-3), and *Copiparvovirus* (PPV4-6) [10]. Recently, a new species of the PPV genotypes, PPV7, was first detected in healthy adult pigs in the USA, and the complete genome sequence of PPV7 isolate 42 was obtained [11]. The amino acid similarity of the NS1 protein between PPV7 and other porcine parvoviruses is less than 30%. Therefore, a new genus, *Chapparvovirus*, has been established for PPV7 (over 30% amino acid identity within a genus) [11].

PPV1 is one of the major causative agents of reproductive failure syndromes in pigs and is characterized by infertility, mummified foetuses, early embryonic death, and stillbirths [12]. This virus is also known to contribute to the development of porcine circovirus-associated disease (PCVAD) [13,14]. PPV6 was first identified in aborted pig foetuses in China in 2014 and was subsequently reported to be co-infected with porcine reproductive and respiratory syndrome virus (PRRSV) in the USA [15,16]. The impact of other PPVs on pig health remains unknown. However, recent research has indicated an association of PPV2, PPV4 and PPV6 with PCV2 infection [8,14]. Furthermore, the presence of PPV4 and PPV6 was detected in foetal tissues [15]. PPV is considered to be a co-factor of PCV2, and concurrent infection with PCV2 and PPV increases disease and lesion severity compared to mono-infection with PCV2 [17,18]. Previous studies have reported PPV3 and PCV2 co-infections in Chinese swine populations and PPV2 and PPV4 co-infection in wild boars in Europe [19]. Recent studies report that at least 3 countries have found PPV7 in their porcine populations, including America, Poland and Korea [11,20,21]. In China, PPV7 was first reported in Guangdong and Anhui provinces in 2017 [22,23]. Interestingly, the PPV7 prevalence of 65.5% on PCV2-positive farms was significantly higher than on PCV2-negative farms, indicating that PPV7 might be associated with PCV2 infection [23].

The purpose of this study was to evaluate the prevalence and diversity of PPV7 in Guangxi, China. The availability of novel porcine parvoviruses allowed us to conduct a comprehensive genetic evolution analysis based on the NS1 and Cap proteins and examine the diversification of these novel viruses.

## Materials and methods

### Sample collection

**Fig 1:**
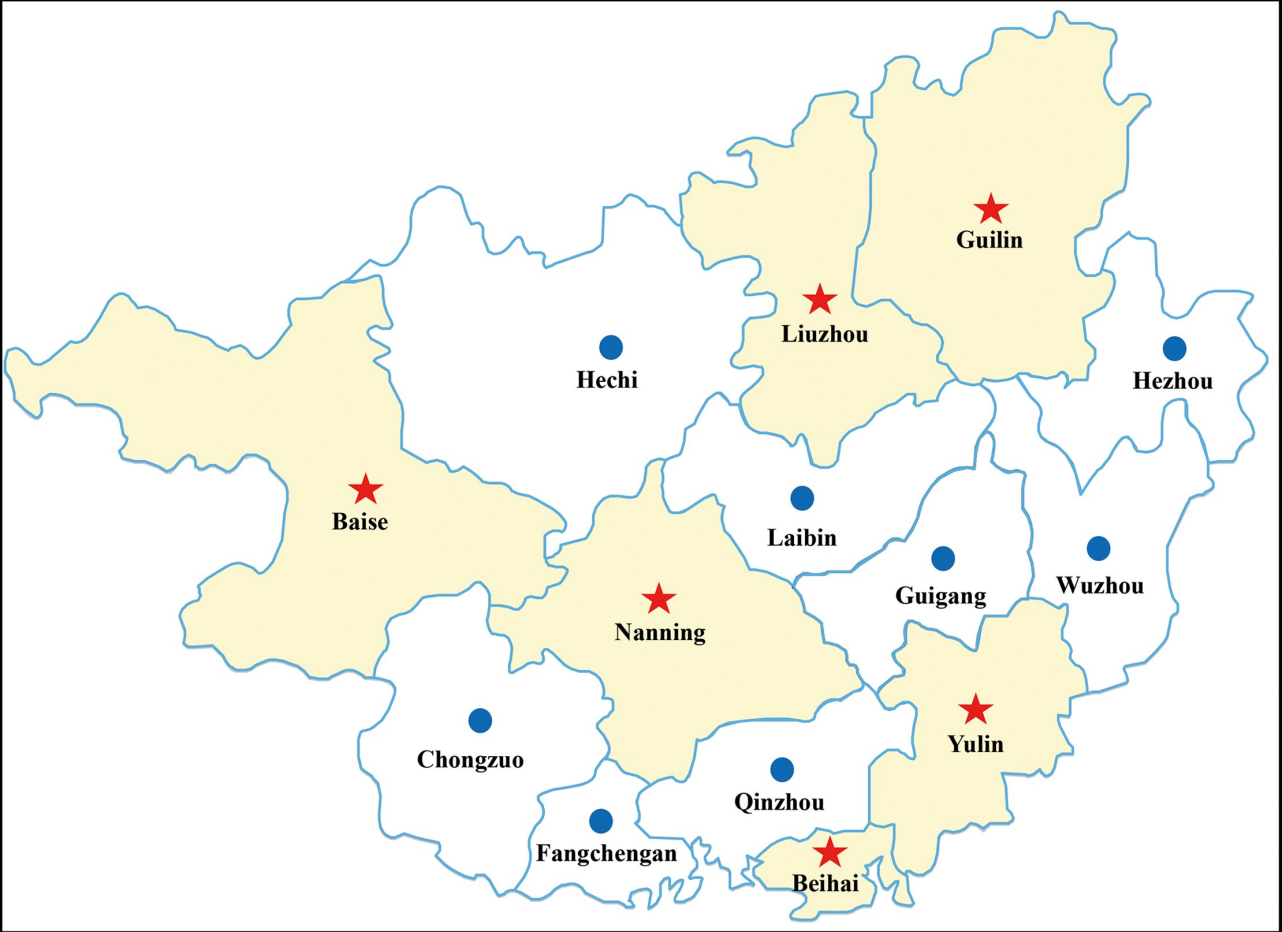
Geographical information for serum samples collected in Guangxi, China. Red stars indicate the geographical location of the sample.

From 2015 to 2017, serum samples (n = 385) of pigs were collected from 11 pig farms in Guilin (N25°17′, E110°17′), Baise (N23° 54′, E106° 36′), Yulin (N22° 39′, E110° 10′), Nanning (N22° 49′, E108° 21′), Liuzhou (N24° 19′, E109° 24′) and Beihai (N21° 29′, E109° 06′) in Guangxi, China (Fig 1). Serum samples used in this study were obtained from the Guangxi Center for Animal Disease Control and Prevention and stored at −20°C. The experiment was approved by the Animal Welfare and the Animal Experimental Ethical Committee (Guangxi University, No. Xidakezi2000138).

### DNA extraction and polymerase chain reaction (PCR)

Total DNA was isolated from tissue samples using the TIANamp Genomic DNA Kit (Tiangen Biotech, China). Four primer pairs were designed based on the reference sequences of isolate 42 (GenBank No. KU563733), and published primers and protocols were used to detect PCV-2 and PPV6 (Table 1). The PCR mixture contained 2 μL of extracted DNA, 2 μL of primer pairs (10 μM), 25 μL of 2×Phanta Max Master Mix (Vazyme, Nanjing, China), and 21 μL of DNase/RNase-free water. The PCR amplification conditions were as follows: predenaturation for 3 minutes at 95°C, followed by 35 cycles of 15 seconds at 95°C, 15 seconds at 62°C, an extension for 1 minute at 72°C, and a final extension for 5 minutes at 72°C. Subsequently, the PCR products were separated using 1.2% agarose gel electrophoresis and cloned into a pMD18-T vector (Takara Co. Dalian). The recombined vectors were amplified in *Escherichia coli* (*E*. *coli*, *DH5α*) for sequencing.

**Table 1:** List of primer sequences used in this study.

Primer	Sequence (5’-3’)	Amplicon length (bp)
PPV7-30-F	GGAACGACAAGGACGACACTT	504
PPV7-533-R	CTTGAGGCTCTGGTATCTTATTGC	
PPV7-417-F	AGCGGGTTCACGGTGGGTAATGCTCTGGG	1320
PPV7-1736-R	TGATGGGTGTTCTCGGCAGGT	
PPV7-1692-F	CGGCCAAGTACAAGAAACCGCAGGACCT	1452
PPV7-3158-R	GGCCAGGTTGTGCCTGCTGTTGGATACG	
PPV7-3115-F	CCGTATCCAACAGCAGGCACAACCTGGCCACA	899
PPV7-4013-R	TGGCGTTGAGAAGACACTGGTTTAG	
PCV-2-F	GGACCCCAACCACATAAAA	555
PCV-2-R	CCCTAACCTATGACCCCTATGT	

### Phylogenetic analysis

Sequences were assembled using SeqMan software (DNASTAR Inc., Madison, Wisconsin, USA) and aligned using MegAlign (DNASTAR Inc., Madison, Wisconsin, USA) with the Clustal W alignment method for genomic similarity analysis. The phylogenetic tree was calculated using the maximum likelihood method (LG+G+I model) with 1,000 bootstrap replicates and constructed on the aligned data set using the MEGA7 program.

## Results

### Detection of PPV7 and PCV2

PPV7 was detected in the six cities. The positive rates of PPV7 and PCV2 in these samples were 27.3% (105/385) and 36.4% (140/385), respectively. The co-infection rate of PPV7 and PCV2 was 17.4% (67/385). Interestingly, the positive rate of PPV7 ranged from 16.3 to 33.3%, with the highest rate recorded in Liuzhou, and the lowest in Yulin (Table 2).

**Table 2:** Frequency and distribution of PPV7 and PCV2 detected by PCR in samples from six cities in Guangxi, China.

Prefecture	Number	PPV7	PCV2	Co-infection
Guilin	57	15	24	11
Baise	34	9	17	5
Yulin	49	8	6	4
Nanning	104	29	26	19
Liuzhou	63	21	18	11
Beihai	78	23	39	17
Total(%)	385	105(27.3%)	140(36.4%)	65(17.4%)

### Multiple sequence alignment and phylogenetic analysis

Seventeen nearly complete PPV7 genome sequences were amplified by PCR. The two major ORFs, ORF1 (encoding NS1) and ORF2 (encoding Cap), were identified in the 17 sample sequences. Based on nucleotide similarity analysis of the complete coding region, the 17 sequences shared 94.1%-100% similarity, with 94.8%-100% similarity in the *NS1* gene and 90.3%-100% similarity in the *cap* gene. In addition, the 17 sample sequences shared 93.9%-97.9% similarity in NS1 and 87.4%-95.0% similarity in the *cap* gene compared with the reference strain.

**Fig 2:**
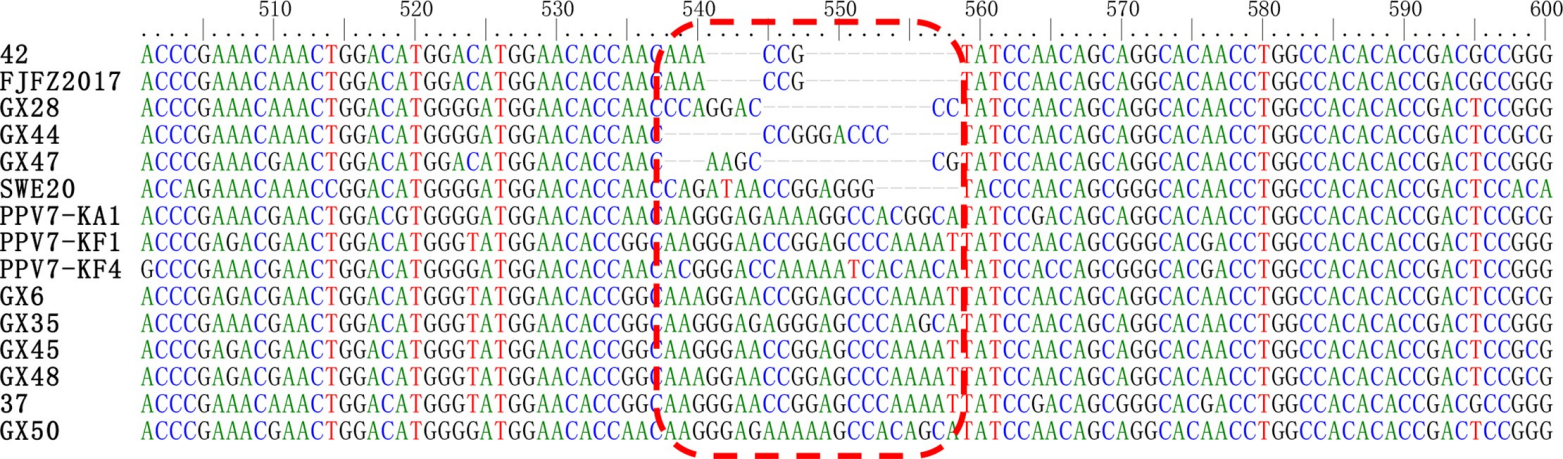
Alignment of nucleotide acid sequences of nucleotide acids representing different isolates of PPV7. The red box highlights the sequence region where additional nucleotides were identified.

Of note, the PPV7 *cap* gene has a length of 1410 nt or 1401 nt; however, in this study, 14 strains with a *cap* region of 1425 nt and two sequences (Gx28 and Gx44) with a *cap* length of 1413 nt were identified. Only one strain (Gx47) was found to have a *cap* gene with a length of 1410 nt. Based on these findings, the sequences in our study contained an additional 3 to 15 nucleotides in the middle of the *cap* gene (Fig 2).

**Fig 3:**
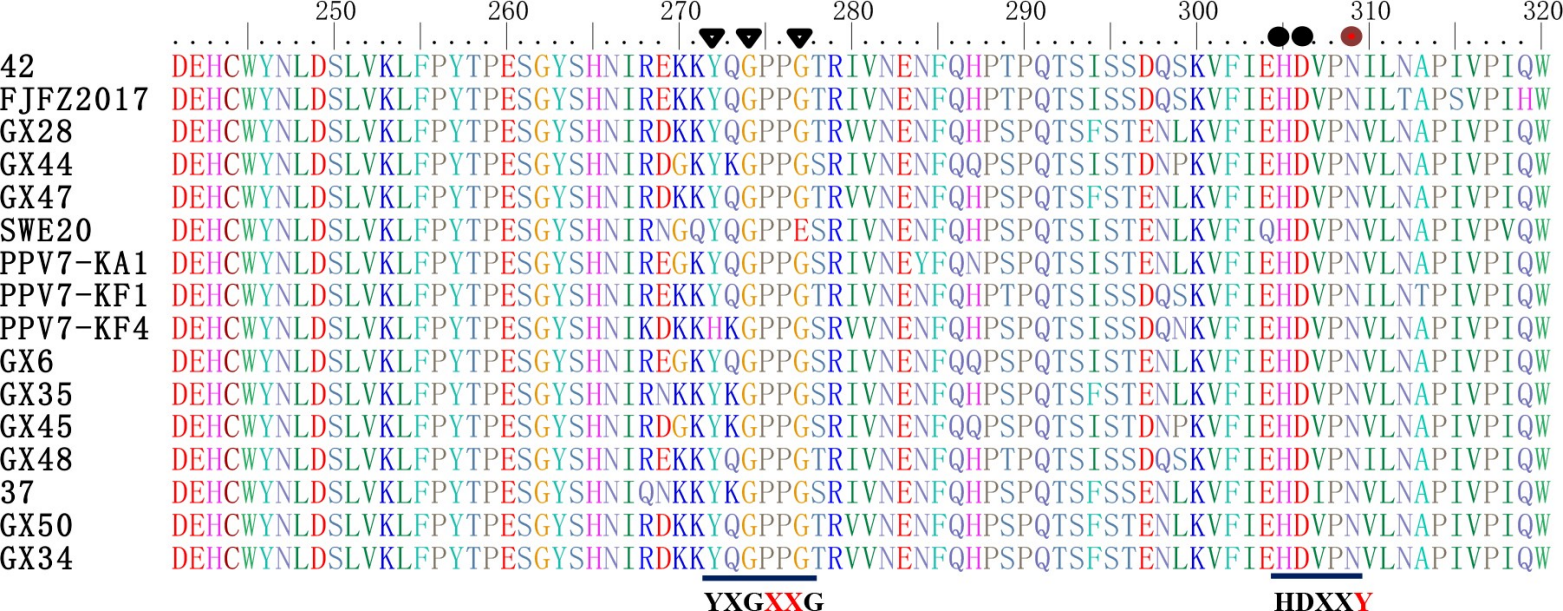
Sequence alignment of the putative phospholipase A2 motif of PPV7 with other parvoviruses. The conserved amino acids of the Ca^2+^ binding loop (YXGXXG) and the catalytic residues (HDXXY) are indicated at the bottom of the alignment. Black circles represent the conserved amino acids of the catalytic residue, and brown circles represent the amino acid mutation sites of the catalytic residues. Black triangles represent the conserved amino acids of the Ca^2+^ binding loop.

The Ca^2+^ binding loop (YXGXG) is present in the capsid proteins of PPV1, PPV2, PPV3 and PPV5 [2,9]. The amino acid sequence of the Ca^2+^ binding loop was “YXGXR” in PPV6 [15]. However, Ca^2+^ binding loops are absent in PPV4. In this study, the conserved amino acid sequence of the Ca^2+^ binding loop is the “YXGXXG” motif in PPV7, rather than the “YXGXR” or “YXGXG” motif found in other parvoviruses (Fig 3). On the other hand, a single amino acid mutation was present at 304 aa (Y to N) in the VP1 protein of all PPV7 strains. Therefore, the catalytic residues (HDXXY) of the putative secretory phospholipase A2 (PLA2) are lacking in PPV7 [9].

**Fig 4:**
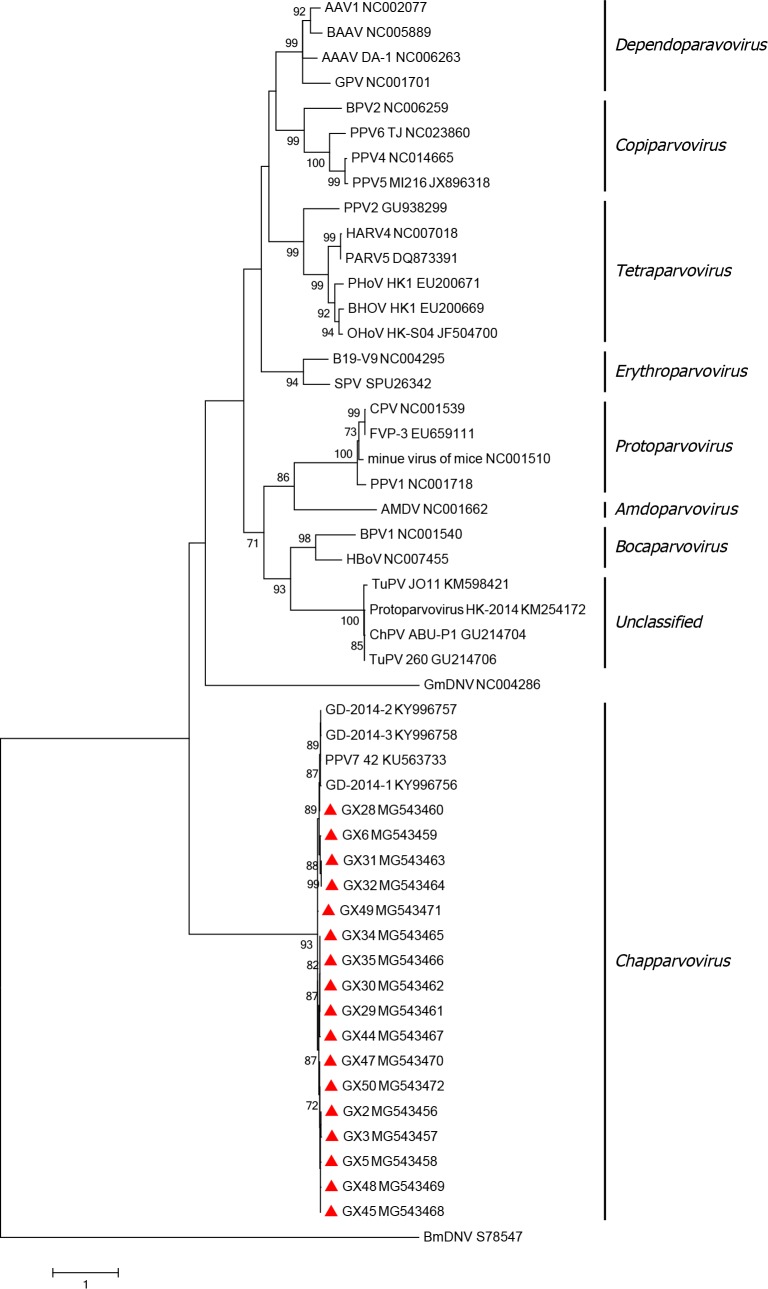
Phylogenetic analysis of viruses in the Parvoviridae family. Phylogenetic reconstruction of amino acid sequences of NS1 by the ML method with the LG+F+I model of sequence evolution with 1,000 bootstrap resampling. The strains identified in this study are represented with a triangle (▲). Scale bars indicate the number of substitutions per site. Only bootstrap support values of >70% are indicated.

To better understand the genetic relationship between the strains identified in this study, a phylogenetic tree was constructed using the maximum likelihood method comparing the NS1 amino acid sequences from our strains and 33 reference strains of *Parvoviridae* family members downloaded from GenBank. Phylogenetic analyses of the amino acid sequences of NS1 revealed that all strains used in this study were in the same branch as PPV7 isolate 42, with all strains belonging to the *Chapparvovirus* genus (Fig 4).

## Discussion

A high level of PCV2 and PPV co-infection in pigs is common in most pig-producing countries [8]. Previous reports revealed that the prevalence of PPV1 ranges from 25.8% to 71.88% [8,17,24]. PPV6 was reported to be co-infected with multiple viruses and associated with abortion in pregnant sows [13,14]. Recently, a new species of the Parvoviruses genus, PPV7, was discovered in rectal swabs from adult pigs [11] and subsequently in Poland and Korea [20,21]. In addition, this virus has become prevalent in Guangdong and Anhui provinces in China [23]. PPV2 and PCV2 are commonly present with PPV7. In this study, we noted a higher PPV7 prevalence in serum samples than in other studies.

PPV7 is 4103 nt in length and contains two major ORFs encoding proteins 672 and 469 amino acids in length [11]. In this study, we noted that the majority of the isolates contained additional nucleotides in the middle of the *cap* gene. Sequence comparison revealed that within nucleotide residues 541–557 at the 5’ end of the *cap* gene, 14 strains had an additional 15 nucleotides, while two strains had an additional three nucleotides, leading to five additional amino acids (within residues 181–186) or one additional amino acid (within residues 181–182). Because of the increased number of amino acids, it may have an effect on the structure and function of the protein. Therefore, the influence of this change on PPV7 requires further study.

Parvoviruses are rapidly evolving viruses with high sequence diversity [2,25]. Frequent recombination between different parvoviruses has long been observed [26]. Several novel porcine parvoviruses have already spread worldwide and show some geographic variation [2,8]. To further study porcine parvoviruses, several studies have attempted to establish cell culture models for virus propagation in different cell types, including porcine kidney (PK-15 and PK-13) cells, swine testicular cells and African green monkey kidney (Vero) cells [15,27,28]. Unfortunately, PPV7 has not yet been successfully isolated.

PCV2 is the main causative agent of PCVAD [29]. Co-infection with PCV2 and other viruses (for example, PCV3, PPV or PRRSV) [18], may lead to a secondary infection following the PCV2-induced depletion of lymphocytes and aggravate clinical symptoms [30]. Some studies have found that co-infection with PCV2 and PPV4 causes more severe disease and lesions than pigs infected with PCV2 alone [14,18]. Allan etc. suggested that PPV-induced immune dysfunction promotes enhanced replication of PCV2 [14]. In this study, nearly one-third of clinical samples were PPV7-positive. Interestingly, the PCV2-positive rate was significantly higher in the PPV7-positive samples than in the non-PPV7 samples, and the difference was extremely significant (P<0.01). The results suggest that PPV7 is likely a significant co-factor in porcine circovirus-associated disease; however, further investigation is still needed to confirm this. PCV2 and PPV contribute to severe disease. Further research is needed to determine if there is any clinical significance associated with novel porcine PPV7 infection.

## Conclusion

In this study, we investigated the prevalence of PPV7 in Guangxi province and conducted genome sequencing of the PPV7 strains found in this province. The high prevalence of PPV7 and high co-infection rate with PCV2 suggests that PPV7 might be co-transmitted with PCV2. Analysis of the Cap protein showed that the protein has significant variability compared with the reference isolate. To date, the number of studies focused on PPV7 is limited. Co-infection with PCV2 and the effects of Cap protein mutations on the virus should be considered in subsequent studies.

## Supporting information

S1 Table Summary of PPV7 identified in the present study. (DOCX)

S2 Table Summary of reference sequence used in this study. (DOCX)

S3 Table Nucleotide similarity analysis. (A) Homologies of the complete sequences of PPV7 isolates. (B) Homologies of the *NS1* gene of PPV7 isolates. (C) Homologies of the *cap* gene of PPV7 isolates.
(DOCX)
