# Supplementary material for: Sequence and phylogenetic analysis of novel porcine parvovirus 7 isolates from pigs in Guangxi, China
Source: PLoS One. 2019 Jul 10;14(7):e0219560. doi: 10.1371/journal.pone.0219560 (PMC6619813; doi:10.1371/journal.pone.0219560)
Supplement: S1 Table — (DOCX) [file pone.0219560.s001.docx]

**S1 Table** Summary of PPV7 identified in the present study

| Number | Name | Host | GenBank accession number |
| --- | --- | --- | --- |
| 1 | GX2 | Pig | MG543456 |
| 2 | GX3 | Pig | MG543457 |
| 3 | GX5 | Pig | MG543458 |
| 4 | GX6 | Pig | MG543459 |
| 5 | GX28 | Pig | MG543460 |
| 6 | GX29 | Pig | MG543461 |
| 7 | GX30 | Pig | MG543462 |
| 8 | GX31 | Pig | MG543463 |
| 9 | GX32 | Pig | MG543464 |
| 10 | GX34 | Pig | MG543465 |
| 11 | GX35 | Pig | MG543466 |
| 12 | GX44 | Pig | MG543467 |
| 13 | GX45 | Pig | MG543468 |
| 14 | GX48 | Pig | MG543469 |
| 15 | GX47 | Pig | MG543470 |
| 16 | GX49 | Pig | MG543471 |
| 17 | GX50 | Pig | MG543472 |
