# Supplementary material for: Sequence and phylogenetic analysis of novel porcine parvovirus 7 isolates from pigs in Guangxi, China
Source: PLoS One. 2019 Jul 10;14(7):e0219560. doi: 10.1371/journal.pone.0219560 (PMC6619813; doi:10.1371/journal.pone.0219560)
Supplement: S2 Table — (DOCX) [file pone.0219560.s002.docx]

**S2 Table** Summary of reference sequence used in this study

| Number | Name | Host | GenBank accession number |
| --- | --- | --- | --- |
| 1 | AAV1 |  | NC002077 |
| 2 | BAAV | Bovine | NC005889 |
| 3 | AAAV | Avian | NC006263 |
| 4 | GPV | Goose | NC001701 |
| 5 | BPV2 | Bovine | NC006259 |
| 6 | PPV6 | Pig | NC023860 |
| 7 | PPV4 | Pig | NC014665 |
| 8 | PPV5 | Pig | JX896318 |
| 9 | PPV2 | Pig | GU938299 |
| 10 | HARV4 | Human | NC007018 |
| 11 | PARV5 | Human | DQ873391 |
| 12 | PHoV | Pig | EU200671 |
| 13 | BHOV | Bovine | EU200669 |
| 14 | OHoV | sheep | JF504700 |
| 15 | B19-V9 | Hunam | NC004295 |
| 16 | SPV | Simian | U26342 |
| 17 | CPV | Dog | NC001539 |
| 18 | FVP | Cat | EU659111 |
| 19 | MVM | Mice | NC001510 |
| 20 | PPV1 | Pig | NC001718 |
| 21 | AMDV | Mink | NC001662 |
| 22 | BPV1 | Bovine | NC001540 |
| 23 | HBoV | Primate | NC007455 |
| 24 | TuPV JO11 | Meleagris gallopavo | KM598421 |
| 25 | Protoparvovirus | Chicken | KM254172 |
| 26 | ChPV ABU-P1 | Chicken | GU214704 |
| 27 | TuPV 260 | Turkey | GU214706 |
| 28 | GmDNV | Galleria mellonella | NC004286 |
| 29 | PPV7(GD-2014-1) | Pig | KY996756 |
| 30 | PPV7(GD-2014-2) | Pig | KY996757 |
| 31 | PPV7(GD-2014-3) | Pig | KY996758 |
| 32 | PPV7 (42) | Pig | KU563733 |
| 33 | PPV7 (37) | Pig | MG902949 |
| 34 | BmDNV | silkworm | S78547 |
