# Supplementary material for: Sequence and phylogenetic analysis of novel porcine parvovirus 7 isolates from pigs in Guangxi, China
Source: PLoS One. 2019 Jul 10;14(7):e0219560. doi: 10.1371/journal.pone.0219560 (PMC6619813; doi:10.1371/journal.pone.0219560)
Supplement: S3 Table — (A) Homologies of the complete sequences of PPV7 isolates. (B) Homologies of the NS1 gene of PPV7 isolates. (C) Homologies of the cap gene of PPV7 isolates. (DOCX) [file pone.0219560.s003.docx]

**S3 Table** Nucleotide similarity analysis. (A) Homologies of the complete sequences of PPV7 isolates. (B) Homologies of the *NS1* gene of PPV7 isolates. (C) Homologies of the *cap* gene of PPV7 isolates.

A. Homologies of the complete sequences of PCV3 isolates

|  | 1 | 2 | 3 | 4 | 5 | 6 | 7 | 8 | 9 | 10 | 11 | 12 | 13 | 14 | 15 | 16 | 17 | 18 | 19 | 20 | 21 | 22 |  |
| --- | --- | --- | --- | --- | --- | --- | --- | --- | --- | --- | --- | --- | --- | --- | --- | --- | --- | --- | --- | --- | --- | --- | --- |
| 1 | *** | 91.4 | 83.8 | 82.9 | 82.8 | 91.4 | 91.5 | 91.4 | 91.7 | 92.8 | 91.1 | 90.7 | 91.8 | 91.8 | 91.3 | 91 | 90.4 | 90.8 | 92.8 | 91.5 | 91.5 | 91.8 | 42 |
| 2 | 6.2 | *** | 80.7 | 80 | 79.8 | 94.9 | 95 | 95.2 | 94.8 | 93.9 | 94.2 | 95 | 94.7 | 94.7 | 94.1 | 94.8 | 93.5 | 95 | 94.2 | 94.7 | 95.1 | 94.8 | 37 |
| 3 | 0.3 | 6.9 | *** | 98.6 | 98.5 | 81.5 | 81.6 | 81.5 | 81.7 | 82.9 | 81.1 | 80.8 | 81.9 | 81.9 | 81.4 | 81.1 | 80.6 | 80.9 | 82.8 | 81.5 | 81.6 | 81.9 | GD-2014-1 |
| 4 | 1.1 | 7.6 | 1.4 | *** | 99.8 | 80.7 | 80.9 | 80.8 | 81 | 82.1 | 80.3 | 80 | 81.2 | 81.2 | 80.6 | 80.4 | 79.9 | 80.2 | 82.1 | 80.8 | 80.9 | 81.1 | GD-2014-2 |
| 5 | 1.3 | 7.9 | 1.6 | 0.2 | *** | 80.6 | 80.7 | 80.6 | 80.8 | 81.9 | 80.2 | 79.9 | 81 | 81 | 80.5 | 80.2 | 79.7 | 80 | 81.9 | 80.6 | 80.7 | 80.9 | GD-2014-3 |
| 6 | 5.8 | 5 | 6.4 | 7.1 | 7.3 | *** | 99.4 | 99.2 | 96.6 | 96.3 | 95.7 | 97.4 | 98.3 | 98.2 | 96.6 | 96.6 | 95.1 | 97.2 | 97.2 | 97.4 | 95.5 | 97.6 | GX2 |
| 7 | 5.6 | 5 | 6.2 | 6.8 | 7.1 | 0.7 | *** | 99.2 | 96.6 | 96.6 | 95.5 | 97.2 | 98.2 | 98.2 | 96.4 | 96.4 | 94.9 | 97.2 | 97.3 | 97.5 | 95.6 | 97.7 | GX3 |
| 8 | 5.8 | 4.8 | 6.3 | 7 | 7.2 | 0.9 | 0.8 | *** | 96.4 | 96.3 | 95.7 | 97.4 | 98.1 | 98.1 | 96.6 | 96.6 | 95.1 | 97.4 | 97.7 | 97.8 | 95.7 | 97.9 | GX5 |
| 9 | 5.5 | 5.2 | 6 | 6.7 | 6.9 | 3.6 | 3.6 | 3.8 | *** | 95.7 | 95.9 | 96.4 | 97.3 | 97.4 | 94.9 | 96.5 | 95.1 | 96.6 | 95.3 | 96.1 | 96.2 | 95.8 | GX6 |
| 10 | 4.4 | 5.9 | 4.8 | 5.5 | 5.8 | 3.6 | 3.3 | 3.6 | 4.3 | *** | 94.7 | 94.9 | 96.8 | 96.8 | 95.5 | 94.9 | 95.4 | 94.7 | 96.5 | 94.6 | 95.7 | 96.7 | GX28 |
| 11 | 6.1 | 5.8 | 6.9 | 7.6 | 7.8 | 4.6 | 4.8 | 4.6 | 4.3 | 5.4 | *** | 97.5 | 94.7 | 94.7 | 99 | 97.6 | 96.7 | 95.5 | 95.8 | 95.3 | 96.5 | 96.8 | GX29 |
| 12 | 6.5 | 5 | 7.2 | 8 | 8.2 | 2.8 | 3 | 2.8 | 3.8 | 5.1 | 2.6 | *** | 96.3 | 96.3 | 97.4 | 98.4 | 96.8 | 97 | 96.2 | 96.6 | 95.7 | 96.5 | GX30 |
| 13 | 5.3 | 5.3 | 5.8 | 6.5 | 6.8 | 1.8 | 1.9 | 2 | 2.8 | 3.1 | 5.7 | 3.9 | *** | 100 | 95.5 | 95.6 | 94.1 | 96.2 | 96.2 | 96.5 | 95.6 | 96.7 | GX31 |
| 14 | 5.3 | 5.3 | 5.8 | 6.5 | 6.7 | 1.8 | 1.9 | 2 | 2.8 | 3.1 | 5.7 | 3.9 | 0 | *** | 95.6 | 95.6 | 94.1 | 96.2 | 96.1 | 96.5 | 95.6 | 96.6 | GX32 |
| 15 | 5.8 | 6 | 6.5 | 7.2 | 7.4 | 3.6 | 3.8 | 3.6 | 5.4 | 4.5 | 1.1 | 2.7 | 4.8 | 4.7 | *** | 97.5 | 96.3 | 94.8 | 96.6 | 95.3 | 95.9 | 97.7 | GX34 |
| 16 | 6.3 | 5.2 | 6.8 | 7.6 | 7.8 | 3.6 | 3.8 | 3.6 | 3.8 | 5.2 | 2.5 | 1.6 | 4.7 | 4.7 | 2.7 | *** | 97.3 | 96.6 | 96 | 95.9 | 96.3 | 96.6 | GX35 |
| 17 | 7.1 | 6.3 | 7.8 | 8.4 | 8.7 | 4.9 | 5.1 | 4.9 | 4.9 | 4.8 | 3.1 | 3 | 6.1 | 6.1 | 3.7 | 2.5 | *** | 96.7 | 95.3 | 95.8 | 96.2 | 95.4 | GX44 |
| 18 | 6.4 | 5 | 7.1 | 7.8 | 8 | 3 | 3 | 2.7 | 3.6 | 5.4 | 4.9 | 3.2 | 4 | 4 | 5.6 | 3.6 | 3.1 | *** | 95.9 | 98.2 | 96.6 | 96.3 | GX45 |
| 19 | 4.6 | 5.5 | 5.1 | 5.8 | 6 | 2.6 | 2.5 | 2 | 4.6 | 3.5 | 4.1 | 3.7 | 3.7 | 3.7 | 3.2 | 3.9 | 4.9 | 4 | *** | 96.5 | 95.4 | 97.5 | GX47 |
| 20 | 5.6 | 5.3 | 6.3 | 7 | 7.2 | 2.7 | 2.7 | 2.3 | 4.1 | 5.5 | 5 | 3.6 | 3.7 | 3.7 | 5 | 4.3 | 4.2 | 1.8 | 3.4 | *** | 95.8 | 96.1 | GX48 |
| 21 | 5.6 | 4.9 | 6.2 | 6.9 | 7.1 | 4.8 | 4.7 | 4.6 | 4 | 4.3 | 3.8 | 4.6 | 4.7 | 4.7 | 4.3 | 4 | 3.8 | 3.6 | 4.5 | 4.4 | *** | 96.7 | GX49 |
| 22 | 5.3 | 5.2 | 5.8 | 6.6 | 6.9 | 2.5 | 2.4 | 2.2 | 4.5 | 3.2 | 3.4 | 3.7 | 3.5 | 3.5 | 2.5 | 3.5 | 4.6 | 3.9 | 2.2 | 4.2 | 3.5 | *** | GX50 |

B. Homologies of NS1 gene of PPV7 isolates.

|  | 1 | 2 | 3 | 4 | 5 | 6 | 7 | 8 | 9 | 10 | 11 | 12 | 13 | 14 | 15 | 16 | 17 | 18 | 19 | 20 | 21 | 22 |  |
| --- | --- | --- | --- | --- | --- | --- | --- | --- | --- | --- | --- | --- | --- | --- | --- | --- | --- | --- | --- | --- | --- | --- | --- |
| 1 | *** | 95.3 | 99.7 | 99.3 | 99 | 95.5 | 95.8 | 95.6 | 96.6 | 97.9 | 94.9 | 94.8 | 96.3 | 96.4 | 94.9 | 94.9 | 94.7 | 95.6 | 95.7 | 95.4 | 96.6 | 95.8 | 42 |
| 2 | 4.8 | *** | 95 | 94.7 | 94.5 | 96 | 96.1 | 96.5 | 95.7 | 95.8 | 95.6 | 95.5 | 95.5 | 95.4 | 95.6 | 95.4 | 95.4 | 96.1 | 96 | 96 | 96.6 | 96.3 | 37 |
| 3 | 0.3 | 5.2 | *** | 99 | 98.7 | 95.2 | 95.5 | 95.3 | 96.3 | 97.6 | 94.7 | 94.5 | 96 | 96.1 | 94.7 | 94.6 | 94.4 | 95.3 | 95.4 | 95.1 | 96.3 | 95.5 | GD-2014-1 |
| 4 | 0.7 | 5.5 | 1 | *** | 99.8 | 94.9 | 95.2 | 95 | 96 | 97.2 | 94.3 | 94.2 | 95.7 | 95.8 | 94.3 | 94.3 | 94.2 | 95 | 95.1 | 94.8 | 96 | 95 | GD-2014-2 |
| 5 | 1 | 5.8 | 1.3 | 0.2 | *** | 94.7 | 95 | 94.7 | 95.8 | 97 | 94.1 | 93.9 | 95.5 | 95.5 | 94.1 | 94 | 93.9 | 94.7 | 94.9 | 94.6 | 95.7 | 94.8 | GD-2014-3 |
| 6 | 4.7 | 4.1 | 5 | 5.3 | 5.6 | *** | 98.7 | 98.4 | 96.2 | 95.7 | 97.2 | 97.4 | 96.5 | 96.4 | 97.2 | 97.2 | 97.1 | 98.4 | 97.6 | 98.2 | 96.5 | 97.5 | GX2 |
| 7 | 4.3 | 4 | 4.6 | 4.9 | 5.2 | 1.3 | *** | 98.4 | 96.2 | 96.3 | 96.8 | 97 | 96.3 | 96.3 | 96.8 | 96.8 | 96.7 | 98.5 | 97.8 | 98.3 | 96.9 | 97.8 | GX3 |
| 8 | 4.6 | 3.6 | 4.9 | 5.2 | 5.5 | 1.7 | 1.6 | *** | 95.8 | 95.8 | 97.1 | 97.4 | 96.3 | 96.2 | 97.1 | 97.2 | 97.1 | 99 | 98.6 | 99.2 | 97.1 | 98.1 | GX5 |
| 9 | 3.5 | 4.4 | 3.8 | 4.1 | 4.4 | 3.9 | 3.9 | 4.3 | *** | 96.7 | 95.8 | 95.8 | 97.6 | 97.7 | 95.8 | 95.7 | 95.5 | 96.1 | 96.1 | 96 | 96.5 | 96.3 | GX6 |
| 10 | 2.1 | 4.3 | 2.4 | 2.8 | 3.1 | 4.4 | 3.8 | 4.3 | 3.4 | *** | 94.9 | 95 | 96.6 | 96.7 | 94.9 | 94.9 | 94.8 | 95.9 | 95.9 | 95.6 | 96.9 | 96.1 | GX28 |
| 11 | 5.3 | 4.6 | 5.6 | 6 | 6.2 | 2.9 | 3.3 | 2.9 | 4.3 | 5.3 | *** | 99.8 | 95 | 95 | 100 | 99.9 | 99.6 | 96.7 | 97.1 | 97.1 | 96 | 97.3 | GX29 |
| 12 | 5.4 | 4.7 | 5.7 | 6.1 | 6.4 | 2.6 | 3.1 | 2.7 | 4.4 | 5.2 | 0.2 | *** | 95.2 | 95.3 | 99.8 | 99.8 | 99.4 | 96.8 | 97.2 | 97.3 | 96 | 97 | GX30 |
| 13 | 3.8 | 4.7 | 4.1 | 4.4 | 4.7 | 3.6 | 3.8 | 3.8 | 2.4 | 3.5 | 5.2 | 4.9 | *** | 100 | 95 | 95 | 94.9 | 96.4 | 95.5 | 96.3 | 96.8 | 95.5 | GX31 |
| 14 | 3.7 | 4.7 | 4 | 4.4 | 4.6 | 3.7 | 3.8 | 3.9 | 2.4 | 3.4 | 5.2 | 4.9 | 0 | *** | 95 | 95.1 | 95 | 96.4 | 95.4 | 96.3 | 96.7 | 95.5 | GX32 |
| 15 | 5.3 | 4.6 | 5.6 | 6 | 6.2 | 2.9 | 3.3 | 2.9 | 4.3 | 5.3 | 0 | 0.2 | 5.2 | 5.2 | *** | 99.9 | 99.6 | 96.7 | 97.1 | 97.1 | 96 | 97.3 | GX34 |
| 16 | 5.3 | 4.7 | 5.6 | 6 | 6.3 | 2.8 | 3.3 | 2.9 | 4.5 | 5.3 | 0.1 | 0.2 | 5.2 | 5.1 | 0.1 | *** | 99.4 | 96.7 | 97 | 97.1 | 96 | 97.2 | GX35 |
| 17 | 5.5 | 4.7 | 5.8 | 6.1 | 6.4 | 2.9 | 3.4 | 3 | 4.7 | 5.4 | 0.4 | 0.6 | 5.3 | 5.2 | 0.4 | 0.6 | *** | 96.6 | 97.1 | 97 | 96.1 | 97.1 | GX44 |
| 18 | 4.6 | 4 | 4.9 | 5.2 | 5.5 | 1.6 | 1.6 | 1.1 | 4 | 4.2 | 3.4 | 3.3 | 3.7 | 3.7 | 3.4 | 3.4 | 3.5 | *** | 98.6 | 99.2 | 96.8 | 98 | GX45 |
| 19 | 4.5 | 4.1 | 4.8 | 5 | 5.3 | 2.5 | 2.3 | 1.4 | 4 | 4.2 | 2.9 | 2.9 | 4.7 | 4.7 | 2.9 | 3.1 | 3 | 1.5 | *** | 99.1 | 96.8 | 98 | GX47 |
| 20 | 4.7 | 4.1 | 5 | 5.4 | 5.6 | 1.8 | 1.7 | 0.8 | 4.1 | 4.5 | 3 | 2.7 | 3.8 | 3.8 | 3 | 2.9 | 3.1 | 0.8 | 0.9 | *** | 96.7 | 97.8 | GX48 |
| 21 | 3.5 | 3.5 | 3.8 | 4.1 | 4.4 | 3.6 | 3.2 | 2.9 | 3.6 | 3.1 | 4.1 | 4.1 | 3.3 | 3.4 | 4.1 | 4.1 | 4 | 3.3 | 3.2 | 3.4 | *** | 97 | GX49 |
| 22 | 4.3 | 3.8 | 4.7 | 5.1 | 5.4 | 2.5 | 2.3 | 2 | 3.8 | 4 | 2.8 | 3.1 | 4.6 | 4.7 | 2.8 | 2.8 | 3 | 2 | 2.1 | 2.2 | 3.1 | *** | GX50 |

C. Homologies of Cap gene of PPV7 isolates.

|  | **1** | **2** | **3** | **4** | **5** | **6** | **7** | **8** | **9** | **10** | **11** | **12** | **13** | **14** | **15** | **16** | **17** | **18** | **19** | **20** | **21** | **22** |  |
| --- | --- | --- | --- | --- | --- | --- | --- | --- | --- | --- | --- | --- | --- | --- | --- | --- | --- | --- | --- | --- | --- | --- | --- |
| **1** | *** | 90.5 | 99.6 | 97.8 | 97.7 | 91.4 | 91.4 | 91.4 | 90.7 | 92.1 | 91.2 | 90.5 | 91.4 | 91.4 | 92 | 91.3 | 90 | 89.6 | 95 | 91.6 | 90.2 | 92.1 | 42 |
| **2** | 9.1 | *** | 90.1 | 88.4 | 88.3 | 93.6 | 93.6 | 93.6 | 93.5 | 90.9 | 92.3 | 94.7 | 93.6 | 93.6 | 92.2 | 94.2 | 90.6 | 93.5 | 91.5 | 92.7 | 93.1 | 92.9 | 37 |
| **3** | 0.4 | 9.6 | *** | 97.5 | 97.4 | 91 | 91 | 91 | 90.2 | 91.6 | 90.7 | 90.1 | 91 | 91 | 91.6 | 90.9 | 89.6 | 89.2 | 94.6 | 91.2 | 89.8 | 91.8 | GD-2014-1 |
| **4** | 1.6 | 10.8 | 1.9 | *** | 99.9 | 89.3 | 89.3 | 89.3 | 88.5 | 90 | 89 | 88.4 | 89.3 | 89.3 | 89.9 | 89.1 | 88 | 87.6 | 92.9 | 89.6 | 88.1 | 90 | GD-2014-2 |
| **5** | 1.7 | 11 | 2 | 0.1 | *** | 89.2 | 89.2 | 89.2 | 88.4 | 89.8 | 88.8 | 88.2 | 89.2 | 89.2 | 89.8 | 89 | 87.9 | 87.4 | 92.8 | 89.5 | 88 | 89.9 | GD-2014-3 |
| **6** | 8 | 6.7 | 8.5 | 9.8 | 9.9 | *** | 100 | 100 | 95.6 | 95.4 | 93.1 | 96.8 | 100 | 100 | 95.9 | 94.5 | 90.3 | 94.2 | 95.7 | 95.9 | 92 | 96.8 | GX2 |
| **7** | 8 | 6.7 | 8.5 | 9.8 | 9.9 | 0 | *** | 100 | 95.6 | 95.4 | 93.1 | 96.8 | 100 | 100 | 95.9 | 94.5 | 90.3 | 94.2 | 95.7 | 95.9 | 92 | 96.8 | GX3 |
| **8** | 8 | 6.7 | 8.5 | 9.8 | 9.9 | 0 | 0 | *** | 95.6 | 95.4 | 93.1 | 96.8 | 100 | 100 | 95.9 | 94.5 | 90.3 | 94.2 | 95.7 | 95.9 | 92 | 96.8 | GX5 |
| **9** | 8.9 | 6.8 | 9.4 | 10.8 | 10.9 | 4.5 | 4.5 | 4.5 | *** | 92.3 | 95.6 | 96.4 | 95.6 | 95.6 | 92.9 | 96.1 | 92.6 | 95.9 | 92.4 | 95.2 | 94.2 | 93.3 | GX6 |
| **10** | 8 | 8.9 | 8.4 | 9.7 | 9.9 | 3.9 | 3.9 | 3.9 | 7.3 | *** | 93.2 | 93.2 | 95.4 | 95.4 | 95.9 | 92.6 | 94.3 | 90.6 | 96.2 | 91.3 | 92 | 96.1 | GX28 |
| **11** | 8.4 | 8.2 | 8.8 | 10.2 | 10.4 | 7.3 | 7.3 | 7.3 | 4.6 | 6.3 | *** | 94.4 | 93.1 | 93.1 | 97.3 | 94.9 | 92.9 | 93.1 | 92.9 | 91.4 | 96.7 | 96.1 | GX29 |
| **12** | 9.1 | 5.5 | 9.5 | 10.9 | 11.1 | 3.3 | 3.3 | 3.3 | 3.7 | 6.3 | 5.9 | *** | 96.8 | 96.8 | 94 | 96.4 | 92.3 | 96.6 | 93.3 | 94.5 | 94 | 94.8 | GX30 |
| **13** | 8 | 6.7 | 8.5 | 9.8 | 9.9 | 0 | 0 | 0 | 4.5 | 3.9 | 7.3 | 3.3 | *** | 100 | 95.9 | 94.5 | 90.3 | 94.2 | 95.7 | 95.9 | 92 | 96.8 | GX31 |
| **14** | 8 | 6.7 | 8.5 | 9.8 | 9.9 | 0 | 0 | 0 | 4.5 | 3.9 | 7.3 | 3.3 | 0 | *** | 95.9 | 94.5 | 90.3 | 94.2 | 95.7 | 95.9 | 92 | 96.8 | GX32 |
| **15** | 7.4 | 8.3 | 7.9 | 9.1 | 9.3 | 4.3 | 4.3 | 4.3 | 7.5 | 3.3 | 2.8 | 6.3 | 4.3 | 4.3 | *** | 94.3 | 91.2 | 91.4 | 95.6 | 91.7 | 95.4 | 98.8 | GX34 |
| **16** | 8.2 | 6.1 | 8.7 | 10 | 10.2 | 5.7 | 5.7 | 5.7 | 4 | 6.9 | 5.3 | 3.7 | 5.7 | 5.7 | 5.9 | *** | 93.1 | 95.2 | 93.5 | 93.3 | 95.2 | 94.5 | GX35 |
| **17** | 10.3 | 9.2 | 10.8 | 12 | 12.2 | 9.5 | 9.5 | 9.5 | 6.9 | 5.5 | 6.6 | 7.3 | 9.5 | 9.5 | 8.5 | 6.4 | *** | 95.7 | 91.2 | 93 | 94.8 | 91.2 | GX44 |
| **18** | 10.1 | 6.8 | 10.6 | 11.8 | 12 | 6.1 | 6.1 | 6.1 | 4.3 | 9.2 | 7.3 | 3.5 | 6.1 | 6.1 | 9.2 | 4.9 | 3.6 | *** | 90.7 | 96.8 | 95 | 92.2 | GX45 |
| **19** | 5.2 | 7.9 | 5.7 | 6.9 | 7.1 | 3.3 | 3.3 | 3.3 | 6.9 | 3.4 | 6.4 | 5.9 | 3.3 | 3.3 | 3.4 | 5.8 | 8.9 | 8.8 | *** | 91.6 | 91.9 | 96.2 | GX47 |
| **20** | 7.8 | 7.7 | 8.3 | 9.5 | 9.6 | 4.3 | 4.3 | 4.3 | 5 | 8.4 | 9.1 | 5.8 | 4.3 | 4.3 | 8.8 | 7.1 | 6.5 | 3.2 | 7.9 | *** | 93.5 | 92.7 | GX48 |
| **21** | 9.5 | 7.2 | 10 | 11.2 | 11.4 | 8.5 | 8.5 | 8.5 | 6.1 | 7.6 | 3.4 | 6.3 | 8.5 | 8.5 | 4.7 | 4.9 | 4.5 | 5.2 | 7.5 | 6.9 | *** | 94.9 | GX49 |
| **22** | 7.2 | 7.5 | 7.6 | 9 | 9.1 | 3.2 | 3.2 | 3.2 | 7.1 | 3.2 | 4.1 | 5.4 | 3.2 | 3.2 | 1.2 | 5.7 | 8.5 | 8.3 | 2.8 | 7.7 | 5.2 | *** | GX50 |
